# Isolated neurofibroma of the urinary bladder incidentally discovered during cystoscopy

**DOI:** 10.1016/j.eucr.2024.102740

**Published:** 2024-04-22

**Authors:** Michael J. Warn, Jason S. Hasegawa, Stephanie J. Handler

**Affiliations:** a School of Medicine, University of California, Riverside, CA, USA; b Department of Obstetrics and Gynecology, University of California, Riverside, CA, USA

**Keywords:** Case report, Neurofibroma bladder, Isolated neurofibroma, Neurofibromatosis

## Abstract

Isolated neurofibromas of the urinary bladder are rare benign tumors typically associated with neurofibromatosis type 1 (NF-1). Herein highlights a bladder neurofibroma incidentally discovered during cystoscopy following midurethral sling removal in a 61-year-old woman without NF-1 sequela. Despite malignancy concerns due to smoking history, histology confirmed a benign neurofibroma. These tumors differ from NF-1-associated neurofibromas in origin and presentation; they are rare, often asymptomatic, and likely stem from somatic mutations. Conservative management is preferred, with surgical intervention indicated only for obstructive masses.

## Highlights

• Neurofibromas rarely present as an isolated bladder mass in patients without NF-1.
• Masses should be biopsied to rule out serious malignancy.
• Without other NF-1 sequelae, treatment for asymptomatic patients is conservative.

## 1 Introduction

Neurofibroma of the bladder was first reported in 1878, during autopsy.^1^ These tumors arise from the pelvic and bladder nerves, with the urinary bladder being the most common location along the urogenital tract.^2^ Neurofibromas, as well as schwannomas, are a subclass of epithelioid sheath tumors generally considered to be associated with neurofibromatosis type 1 (NF-1).^3^^,^^4^ While these tumors in NF-1 patients comprise less than 0.1 % of all bladder tumors, isolated urinary bladder neurofibromas in patients without NF-1 sequelae are exceedingly rare with less than 10 isolated cases reported to date.^5^, ^6^, ^7^, ^8^, ^9^, ^10^, ^11^

In NF-1, patients frequently present with cutaneous manifestations on physical exam and undergo subsequent disease screening and cross-sectional imaging which often reveals other tumor sites and disease sequelae. However, in cases where neurofibromas are solitary, findings are frequently incidental.^1^^,^^6^^,^^7^ As these tumors are benign based on histologic markers, surgical resection is indicated only for obstructive pathologies, with conservative management otherwise.

## 2 Case presentation

**Fig. 1:**
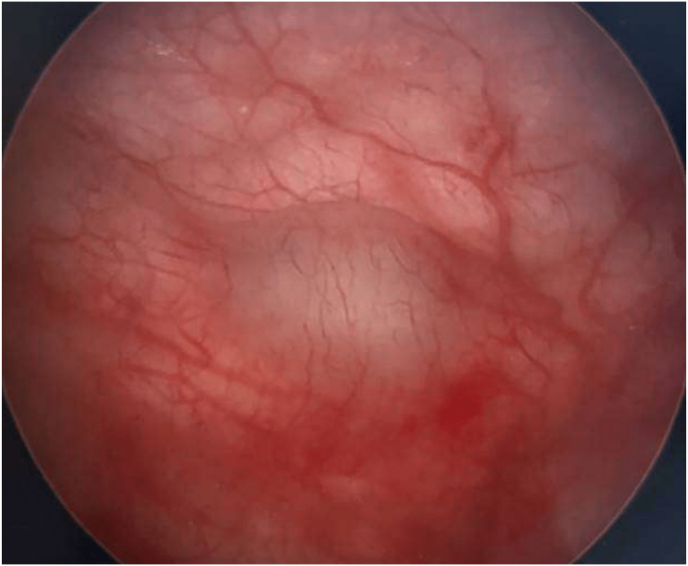
Image of neurofibroma obtained during surgery via cystoscopy.

We report the case of a 61-year-old female who presented to the urogynecology clinic in consultation for recurrent urinary tract infection (UTI), pelvic pain, and voiding dysfunction since she underwent surgical management of stress urinary incontinence via mesh midurethral sling 19 years ago. Social history is relevant for being a current tobacco smoker. Workup for recurrent UTI included two cystoscopies (seven and three years prior to presentation to our clinic), during which neither mesh exposure not bladder masses were observed. She did not report any family history of NF-1, nor display any clinical manifestations such as cutaneous neurofibromas, café-au-lait spots, or Lisch nodules. Pelvic exam revealed palpable mesh in the anterior vaginal wall, but not mesh exposure. The patient was counseled and elected surgical management of her chronic pelvic pain and voiding dysfunction via vaginal removal of midurethral sling, urethrolysis, and cystoscopy. During cystoscopy, a non-obstructing approximately three to four mm solid mass in the midline of posterior bladder wall just cephalad to the trigone was visualized and biopsied (Fig. 1). The tissue was sent for histopathologic analysis which revealed benign spindle cell neoplasm positive for S100, CD34, and Sox-10, with low ki-67 proliferation. Histopathology was most consistent with benign neurofibroma of the bladder. Postoperatively, she did well: her voiding dysfunction resolved, and her chronic pelvic pain drastically improved. No incontinence symptoms were reported at follow up. Subsequent resection of the neurofibroma was not indicated as the tumor did not compress the trigone or urethrovesical junction, and her conditions resolved or improved.

## 3 Discussion

Isolated neurofibromas of the bladder are exceedingly rare and account for fewer than 0.1 % of all bladder tumors.^2^ Typically, neurofibromas are associated with NF-1, an autosomal dominant hereditary germline disease that classically presents with numerous findings including pigmented lesions, skeletal abnormalities, and brain tumors.^3^ A 1999 study of four cases of neurofibroma of the bladder revealed none of the cases resulted in malignant transformation after ten years.^12^ However, other studies have reported malignant transformations in these neurofibroma cases at rates between 5 %^5^ and as high as 29 %,^13^ albeit in association with NF-1 and in plexiform types, respectively. Chengrui et al. recently reported that patients with isolated cutaneous neurofibromas possessed only somatic NF-1 mutations within the tumor without germline mutation or any other disease sequelae.^4^ Given the possibility of differing somatic versus germline origins,^4^ malignant potential of these tumors may depend on disease origin and association. Regardless, because of the broad differential diagnosis of perineuoma, paragalgioma, postoperative spindle cell lesion, and the more serious leiomyosarcoma or urothelial carcinoma, biopsy should be performed in all isolated cases,^9^ especially in those with significant smoking history, such as the patient in this case. Neurofibromas have a very good prognosis and chance of malignant transformations is rare, if any; however, surveillance has been suggested in cases of asymptomatic disease.^3^^,^^4^^,^^7^^,^^14^, ^15^, ^16^

Symptoms from isolated bladder neurofibromas are typically due to mass effect causing dysuria, hematuria, irritation, and trouble voiding or urinary retention from tumors arising near the bladder neck. While the patient in this case had symptoms consistent with voiding dysfunction, this improved with excision of the midurethral sling, suggesting that the sling was causing bladder outlet obstruction. The tumor was not adjacent to the urethrovesical junction; therefore, further resection of the neurofibroma was not indicated.

The vast majority of neurofibromas are thought to be associated with NF-1, and sporadic cases are at least a tenth as rare.^5^, ^6^, ^7^, ^8^, ^9^, ^10^, ^11^ NF-1 genotyping in solitary cutaneous neurofibromas suggested these tumors are likely acquired somatic mutations vs germline.^4^ Whether these cases are a mild or early form of NF-1, or a different etiology altogether, can only be speculated upon given the rarity. Interestingly, tumors associated with autosomal dominant NF-1 tend to arise in a young male demographic.^17^^,^^18^ Although it is possible that this patient’s neurofibroma had been missed on two previous cystoscopies, this may support the notion that the tumor arose via somatic mutation within the last three years since her last cystoscopy.

## 4 Conclusion

The present case details the discovery and conservative management of a rare, isolated neurofibroma in the urinary bladder in an older female patient without a diagnosis, disease sequelae, or family history of neurofibromatosis.

## Provenance and peer review

Not commissioned, externally peer reviewed.

## Ethical approval

Ethical Approval was provided/waived by the author’s institution.

## Patient consent

Verbal informed consent was obtained from the patient for publication of this case report and accompanying images. Written consent is available for review by the Editor-in-Chief of this journal on request.

## Sources of funding

This research did not receive any specific grant from funding agencies in the public, commercial, or not-for-profit sectors.

## CRediT authorship contribution statement

**Michael J. Warn:** Writing – review & editing, Writing – original draft, Validation, Methodology, Investigation, Formal analysis, Data curation, Conceptualization. **Jason S. Hasegawa:** Writing – review & editing, Writing – original draft, Investigation, Data curation. **Stephanie J. Handler:** Writing – review & editing, Writing – original draft, Validation, Supervision, Resources, Project administration, Methodology, Conceptualization.

## Conflicts of interest

None.

## References

[1] Zugail A.S., Benadiba S., Ferlicot S., Irani J. (2017). Oddities sporadic neurofibroma of the urinary bladder. A case report. Urol Case Rep.

[2] Hintsa A., Lindell O., Heikkilä P. (1996). Neurofibromatosis of the bladder. Scand J Urol Nephrol.

[3] Gutmann D.H., Ferner R.E., Listernick R.H., Korf B.R., Wolters P.L., Johnson K.J. (2017). Neurofibromatosis type 1. Nat Rev Dis Prim.

[4] Guo C., Zhou L., Sun Y., Hu X. (2022). How to distinguish solitary neurofibroma from neurofibromatosis type 1. J Craniofac Surg.

[5] Rober P.E., Smith J.B., Sakr W., Pierce J.M. (1991). Malignant peripheral nerve sheath tumor(malignant schwannoma) of urinary bladder in Von Recklinghausen neurofibromatosis. Urology.

[6] Baugh B., Stencel M., Patel A., Hale N. (2020). An isolated case of an incidentally discovered neurofibroma of the urinary bladder. Urol Case Rep.

[7] Umakanthan S., Naik R., Bukelo M.M., Rai S., Prabhu L. (2015). Primary bladder neurofibroma: a rare case with clinical implications and diagnostic challenges. J Clin Diagn Res.

[8] Winfield H.N., Catalona W.J. (1985). An isolated plexiform neurofibroma of the bladder. J Urol.

[9] Wang W., Montgomery E., Epstein J.I. (2008). Benign nerve sheath tumors on urinary bladder biopsy. Am J Surg Pathol.

[10] Hajihashemi A., Geravandi M. (2023). Pelvic neurofibroma in a patient presenting with pelvic pain and urinary frequency: a case report. Radiol Case Rep.

[11] Castillo P.M.C., Gregorio S.A., Alcaide J.R.C., Basan A.A., Jjdlp Barthel (2006). Neurofibroma de la vejiga: Caso clínico y revisión de la literatura. Arch Esp Urol.

[12] Cheng L., Scheithauer B.W., Leibovich B.C., Ramnani D.M., Cheville J.C., Bostwick D.G. (1999). Neurofibroma of the urinary bladder. Cancer.

[13] Clark S.S., Marlett M.M., Prudencio R.F., Dasgupta T.K. (1977). Neurofibromatosis of the bladder in children: case report and literature review. J Urol.

[14] Sørensen S.A., Mulvihill J.J., Nielsen A. (1986). Long-Term Follow-up of von Recklinghausen Neurofibromatosis. N Engl J Med.

[15] Üre I., Gürocak S., Gönül I.I., Sözen S., Deniz N. (2013). Neurofibromatosis type 1 with bladder involvement. Case Rep Urol.

[16] Avery R.A., Katowitz J.A., Fisher M.J. (2017). Orbital/periorbital plexiform neurofibromas in children with neurofibromatosis type 1: multidisciplinary recommendations for care. Ophthalmology.

[17] Ferner R.E. (2007). Neurofibromatosis 1 and neurofibromatosis 2: a twenty first century perspective. Lancet Neurol.

[18] Widemann B.C. (2009). Current status of sporadic and neurofibromatosis type 1-associated malignant peripheral nerve sheath tumors. Curr Oncol Rep.

